# 2-Amino-3-methylcarboxy-5-heptyl-thiophene (TJ191) is a selective anti-cancer small molecule that targets low TβRIII-expressing malignant T-cell leukemia/lymphoma cells

**DOI:** 10.18632/oncotarget.23501

**Published:** 2017-12-15

**Authors:** Ahmed El-Gazzar, Sam Noppen, Joice Thomas, Wim Dehaen, Jan Balzarini, Sandra Liekens

**Affiliations:** ^1^ Rega Institute for Medical Research, Department of Microbiology and Immunology, KU Leuven, 3000 Leuven, Belgium; ^2^ Department of Chemistry, KU Leuven, 3000 Leuven, Belgium

**Keywords:** cytostatic/cytotoxic anti-cancer molecule, T-cell leukemia/lymphoma, TβRIII

## Abstract

Current chemotherapy regimens often include non-specific cytostatic/cytotoxic drugs, which do not distinguish between normal and tumor cells, therefore causing considerable systemic toxicity. We previously reported the synthesis and anti-proliferative activity of a novel synthetic 2-aminothiophene-3-carboxylic acid ester derivative TJ191 that selectively targets certain cancer cells without affecting the proliferation of other cancer cells or normal fibroblasts or immune cells (over 600-fold selectivity). In a panel of ten human T-cell leukemia/lymphoma cell lines and peripheral blood mononuclear cells (PBMCs), we now found that transforming growth factor β type III receptor (TβRIII) expression correlates inversely with TJ191 sensitivity, but not with sensitivity against classical chemotherapeutic drugs, thus serving as a predictive marker for TJ191 sensitivity. Accordingly, CRISPR/Cas9-mediated knock-out of TβRIII partially restored the susceptibility of TJ191-resistant cells to this novel compound. Our findings highlight TJ191 as a potent and selective anti-cancer molecule with pronounced activity against human malignant T-cells expressing low levels of TβRIII.

## INTRODUCTION

Current cancer chemotherapy regimens still mainly rely on the use of non-specific cytostatic/cytotoxic drugs, which cause systemic toxicity and induce resistance, often leading to tumor recurrence and treatment failure [1, 2]. Thus, a new generation of more selective chemotherapeutic agents is urgently needed. In the past two decades, there has been a tremendous increase in our knowledge of the molecular mechanisms and pathophysiology of human cancer [3]. Several of these mechanisms have been exploited as new targets for drug development [4]. However, most tumors are dependent on various signaling and metabolic pathways and are highly heterogeneous. It is therefore unlikely that addressing a single drug target will eliminate cancerous growth. Successful cancer therapy will rather require the development of appropriate drug cocktails, which may include non-specific cytostatic as well as specific (multi) targeted drugs.

Several signaling pathways are genetically and epigenetically altered in cancer, including the transforming growth factor-β (TGF-β) pathway. The TGF-β superfamily controls diverse fundamental biological processes including cell differentiation, proliferation, motility, apoptosis and embryonic development in a cell type- and context-specific manner [5, 6]. TGF-β signaling has a dual function in cancer progression [7]. TGF-β can act as tumor suppressor by inhibiting cell proliferation and genomic instability, and by activating apoptosis and senescence. In contrast, mutated TGF-β signaling can prompt tumor invasiveness and metastasis at a late stage of tumorigenesis by activating angiogenesis, migration, invasion and epithelial-mesenchymal transition (EMT) [7, 8].

TGF-β binds mainly to three cell surface receptors (TβR) including type I (TβRI), type II (TβRII) and type III (TβRIII). The most abundantly expressed TGF-β receptor is TβRIII, also known as betaglycan [9]. TβRIII can bind and present TGF-β to TβRII to augment TGF-β activity [10]. TβRIII has been shown to regulate cell growth, migration, invasion, angiogenesis and EMT in cancer cells. So far, no mutation has been described in the gene encoding TβRIII, *TGFBR3*, but reduced or loss of TβRIII expression has been observed in various cancers [11–13]. Previous studies have shown that ectopic expression of recombinant soluble TβRIII antagonized TGF-β signaling cascades, thereby suppressing both anchorage-dependent and -independent breast cancer cell growth *in vitro* [14]. In addition, administration of soluble TβRIII suppresses angiogenesis, tumor growth and metastasis in a breast cancer mouse model [15].

We previously reported the synthesis and anti-proliferative activity of novel synthetic 2-aminothiophene-3-carboxylic acid ester derivatives [16, 17]. Further structure activity relationship studies led to the design and synthesis of 2-amino-3-methylcarboxy-5-heptyl-thiophene TJ191 [18]. This compound preferentially inhibited the proliferation of cell lines derived from T-cell (but not B-cell) leukemia/lymphoma, but also several renal, liver and prostate cancer cell lines, without affecting normal fibroblasts or immune cells (500–1000-fold selectivity). Tumor selectivity could not be explained by differential cellular drug uptake as experiments using a fluorescent TJ191 derivative demonstrated that both sensitive and insensitive (tumor) cell lines rapidly take up the drug, after which it is predominantly localized in the cytoplasm [18].

In the current study, we further examined the activity of TJ191 against an extended panel of 10 T-cell leukemia/lymphoma cell lines. We showed that TJ191 not only elicits cytostatic effects but also induces apoptosis in sensitive T-cell leukemia cells. Moreover, we identified TβRIII as a determinant of TJ191 sensitivity in T-cell leukemia/lymphoma cells, with high TβRIII expression level corresponding to TJ191 resistance and low TβRIII expression corresponding to sensitization to the TJ191-induced anti-proliferative effects.

## RESULTS

### Cytostatic/cytotoxic effects of TJ191 in T-cell leukemia cell lines

**Figure 1:**
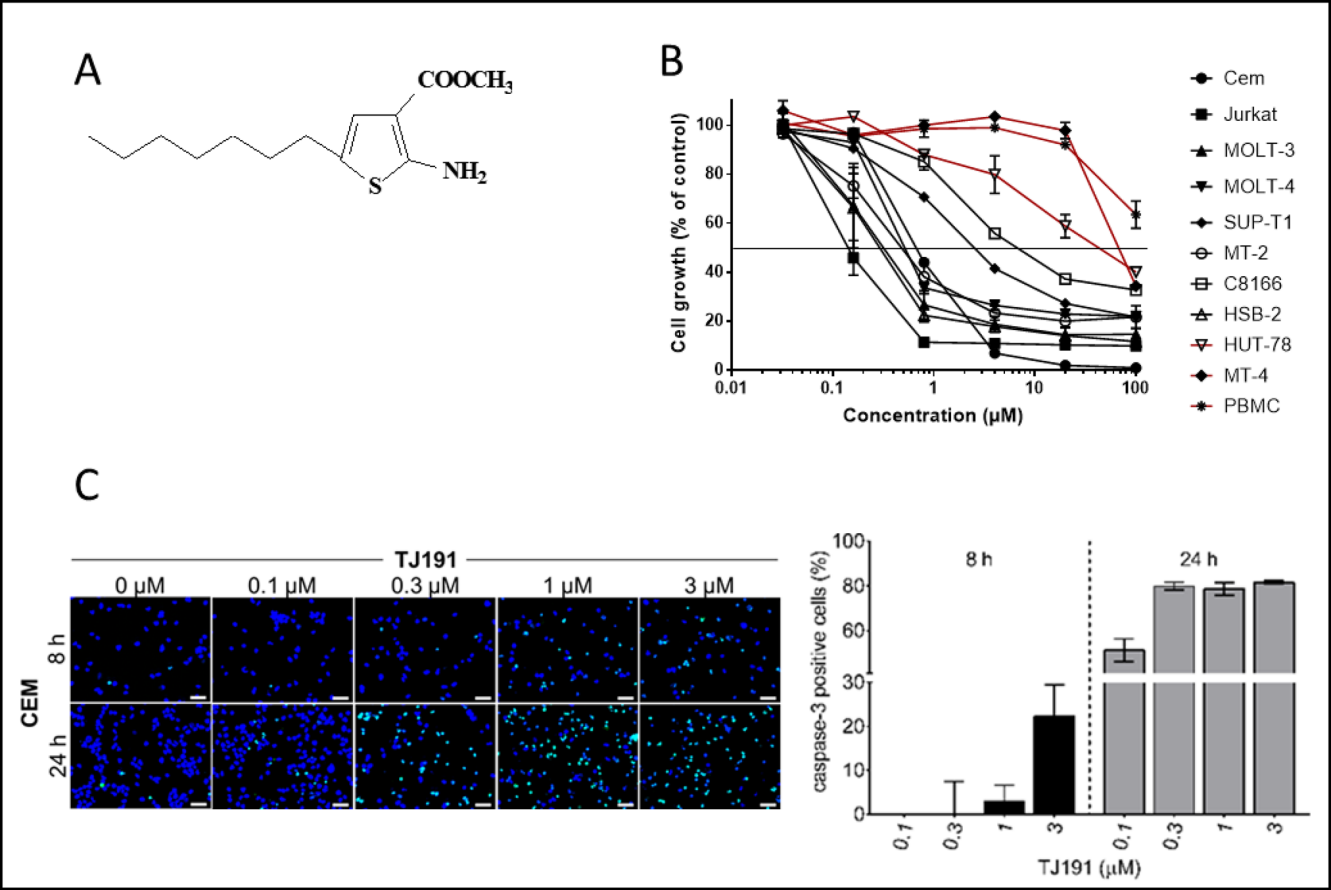
Cytostatic and cytotoxic activity of TJ191 in T-cell leukemia/lymphoma cells (**A**) Chemical structure of TJ191. (**B**) Effect of TJ191 on the growth of human T-cell leukemia/lymphoma cell lines. (**C**) Pro-apoptotic effect of TJ191 in CEM cells. Cells were incubated with TJ191 for 8 h or 24 h and apoptosis was determined based on caspase-3 activity using the NucView 530 Caspase-3 substrate, according to the manufacturer’s instruction, and fluorescence microscopy (Axiovert 200 M inverted microscope, Zeiss). Left panel, representative fluorescence microscopy images are shown; scale bars, 50 µm. Right panel, quantification of the apoptosis rate is shown. Bars represent the mean percentage of cells stained positive for caspase-3 of three different sections; bars, ± S.E.M. Data are representative of two independent experiments.

We recently reported the specific and potent anti-proliferative activity of TJ191 (Figure 1A), in T-cell leukemia/lymphoma cells and various solid tumor cell lines of liver, kidney, lung, breast, ovarian, prostate, central nervous system and colon cancer origin [18]. Interestingly, the growth of primary human fibroblasts or PBMCs was not, or hardly, affected by TJ191 (IC_50_ > 100 µM), resulting in > 600-fold selectivity, *i.e.* IC_50_ of ∼100nM in drug-sensitive versus ∼60 µM in drug-insensitive tumor cell lines [18].

Here, we focused our further analysis on T-cell leukemia and lymphoma, since these malignancies showed the highest response rate to TJ191 among the tested cancer cell types (Figure 1B). In particular, TJ191 exhibited pronounced anti-proliferative activity in CEM (IC_50_ = 0.13 ± 0.02 µM), JURKAT (IC_50_ = 0.13 ± 0.08 µM), MOLT-3 (IC_50_ = 0.26 ± 0.19 µM), MOLT-4 (IC_50_ = 0.22 ± 0.11 µM), SUP-T1 (IC_50_ = 1.5 ± 0.02 µM), MT-2 (IC_50_ = 0.32 ± 0.086 µM), C8166 (IC_50_ = 3.1 ± 0.5 µM) and HSB-2 (IC_50_ = 0.26 ± 0.16 µM), but not in HUT-78 (IC_50_ = 17 ± 10 µM) and MT-4 (IC_50_ = 47 ± 5 µM) cells. Cell counting at the end of the incubation period showed a cytotoxic effect at the higher drug concentrations (i.e. lower cell number than at the start of the experiment). Therefore, we investigated the effect of TJ191 on induction of apoptosis. The sensitive CEM cell line was treated with TJ191 at different concentrations ranging from 0.1 µM to 3 µM for either 8 h or 24 h. Thereafter, the cells were fixed and cleaved caspase-3 activity was analyzed using fluorescence microscopy. TJ191 was capable of mediating apoptosis in a concentration- and time-dependent manner. Even at 0.3 µM, TJ191 could induce the maximum apoptotic rate of ∼80% after 24 h (Figure 1C).

Altogether, these results indicate that TJ191 represents a novel anti-cancer drug with the potential to selectively inhibit the proliferation of, and induce apoptosis in, various T-cell-derived hematological malignant cell lines.

### TβRIII acts as a predictive marker for TJ191 sensitivity in malignant T-cells

**Figure 2:**
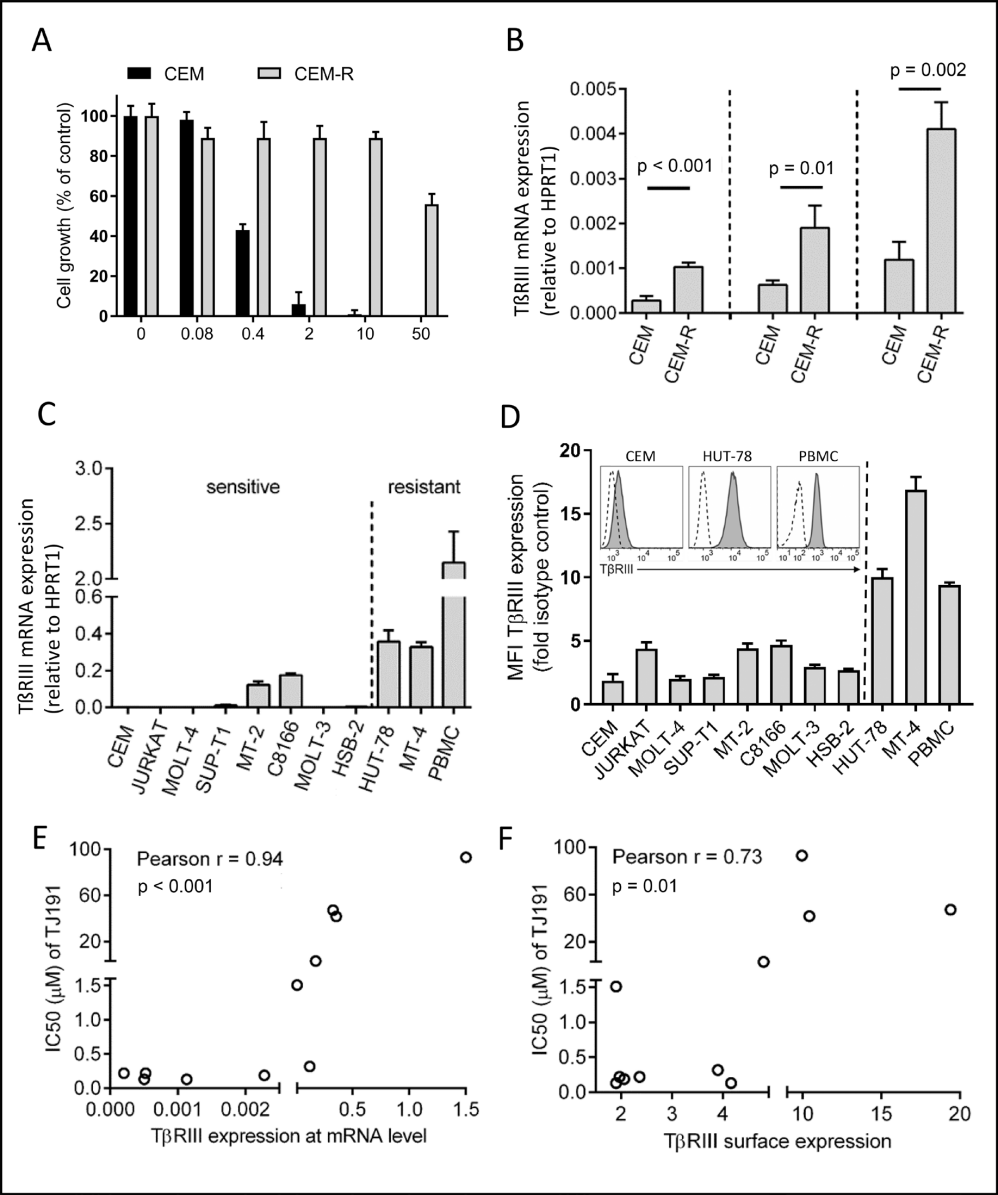
Inverse correlation between TβRIII expression and TJ191 sensitivity in malignant T cells (**A**) Sensitive human CEM cells were grown for one month in the presence of a 100-fold IC_50_ concentration of TJ191 (CEM-R). Afterwards, parental CEM and CEM-R cells were grown in the presence of the specified concentrations of TJ191 for three days and counted. The graph shows the sensitivity of parental and drug-resistant CEM cells to TJ191. Data are representative of three independent experiments. (**B**) mRNA expression levels of TβRIII in sensitive and resistant CEM cells were measured by qRT-PCR. Bars represent the mean of triplicates ± S.E.M. Three independent experiments are shown. (**C**) TβRIII expression was measured by qRT-PCR in a panel of 10 T-cell leukemia/lymphoma cell lines and healthy PBMCs. Bars represent the mean of triplicates ± S.E.M. Data are representative of three independent experiments. (**D**) Expression of TβRIII on the surface of 10 T-cell leukemia/lymphoma cell lines and healthy PBMCs. Mean Fluorescence intensity (MFI) of TβRIII versus isotype control is shown. Inset shows TβRIII expression in TJ191-sensitive CEM and -resistant HUT-78 cells and normal PBMC-derived lymphocytes. Dashed black lines in unfilled profile represent Ig isotype control staining and grey-filled profiles indicate surface TβRIII expression. Data are representative of three independent experiments. (**E** and **F**) Correlations and associated *p*-values between (E) TβRIII mRNA expression or (F) Fold increase in mean fluorescence intensity (MFI) of surface TβRIII expression and IC_50_ of TJ191 in 10 T-cell leukemia/lymphoma cell lines and PBMCs.

To understand the mechanism of action of TJ191, we selected drug-resistant CEM cells by applying a 100-fold IC50 concentration of TJ191 to wild-type CEM cells. Within one month a population, referred to as CEM-R, was selected with ∼100-fold reduced sensitivity to TJ191 (Figure 2A). Next, the gene expression profile of wild-type CEM and CEM-R cells was compared using RNA sequencing. One of the genes that was upregulated in CEM-R cells was the gene that encodes TβRIII (data not shown). Since TβRIII is known to play a pleiotopic role in cancer development, we decided to focus our attention on this receptor. TβRIII mRNA expression level was 3.5-fold increased in CEM-R compared to CEM cells as determined by qRT-PCR (Figure 2B). We then validated TβRIII mRNA expression by qRT-PCR (Figure 2C, 2E) and TβRIII protein expression (Figure 2D, 2F) by flow cytometry in a panel of ten T-cell leukemia and lymphoma cell lines and PBMC samples from four different healthy donors. We found that TβRIII is highly expressed in naturally TJ191-resistant T-cell leukemia/lymphoma cell lines and PBMC-derived lymphocytes (Figure 2D). Moreover, TβRIII expression correlated inversely with TJ191 sensitivity (*r* = 0.94, *p* < 0.001, Figure 2E). A similar correlation was found at the protein level (*r* = 0.73, *p* = 0.01, Figure 2F).

**Figure 3:**
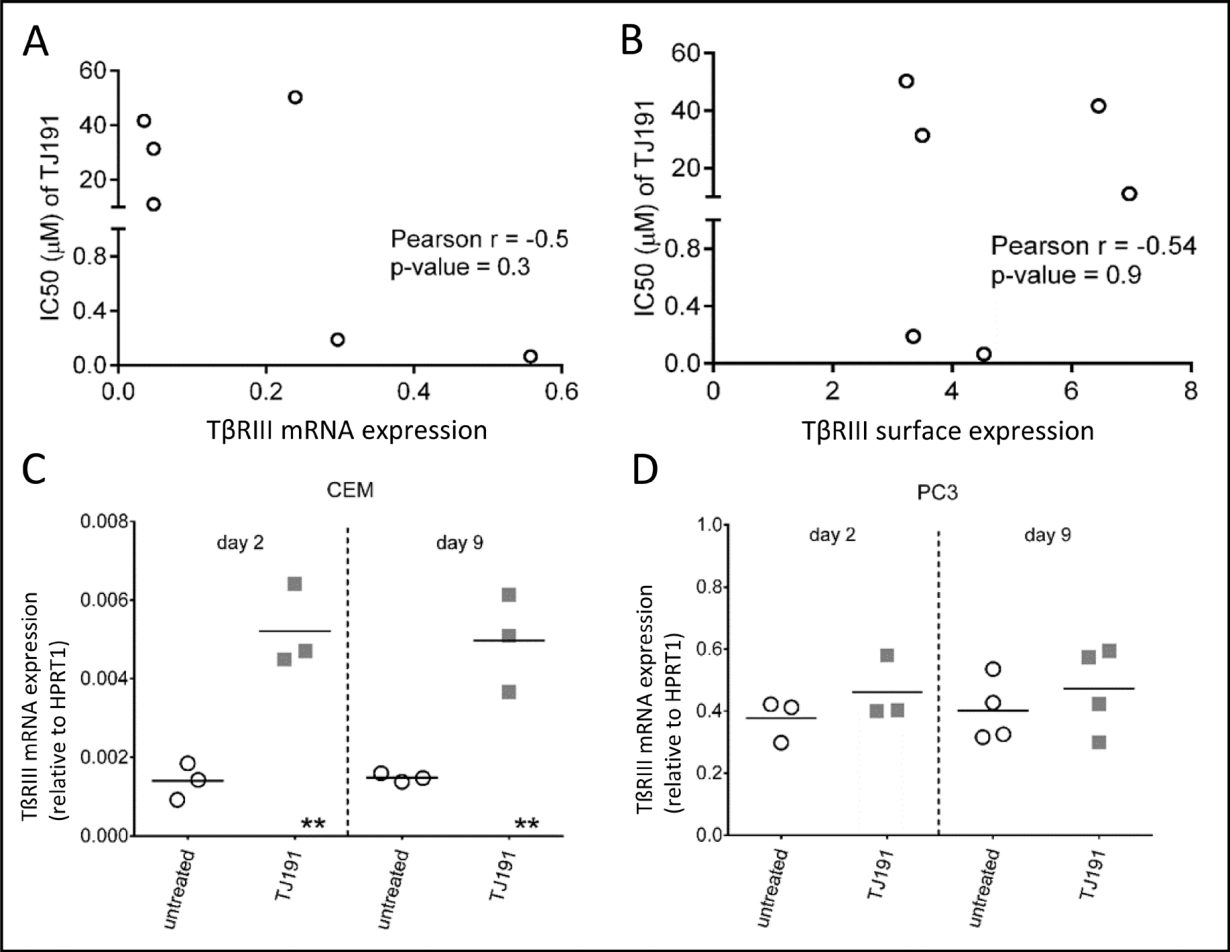
mRNA and surface TβRIII expression in prostate cancer cell lines The IC_50_ of TJ191 was determined in six different prostate cancer cell lines (PC3, BPH-1, DU-145, PNT1A, 22RV1, and LNCAP) and correlated with TβRIII expression on (**A**) mRNA by qRT-PCR, and (**B**) surface levels by flow cytometry. There was no correlation between the IC_50_’s of TJ191 and mRNA or surface TβRIII expression levels. Correlations were determined using the Pearson product-moment correlation coefficient. Data shown were collected from three independent experiments. (**C** and **D**) wild-type C) CEM and D) PC3 cells were left untreated or treated with 10 µM of TJ191 for two or nine days. Remaining TJ191-resistant live cells (∼15–20%) were harvested and TβRIII expression was determined on mRNA level by qRT-PCR. The horizontal line inside each group represents the mean of at least three independent experiments. TβRIII expression increased significantly in TJ191-resistant CEM cells, but not in PC3 cells, when compared with untreated control. ^**^p < 0.005.

Next, we investigated whether this inverse correlation between TJ191 sensitivity and TβRIII expression in T-cell leukemia/lymphoma can be extended to other cancer types. We determined TβRIII expression levels by qRT-PCR and flow cytometry and correlated the values with the IC_50_ values in a panel of 6 prostate cancer cell lines. Remarkably, although the growth of several prostate cancer cell lines was strongly inhibited by the compound, we were unable to find a significant correlation in the tested prostate cancer cell lines at TβRIII mRNA (*r* = -0.5, *p* = 0.3) or cell surface level (*r* = -0.54, *p* = 0.9) (Figure 3A, 3B).

If TβRIII expression is a specific determinant of TJ191 sensitivity in T-cell leukemia/lymphoma cells, we would expect an increase in TβRIII expression level on leukemia cells that remained viable after TJ191 treatment (Figure 1C). Therefore, we treated TJ191-sensitive leukemia CEM and prostate PC3 cells with 10 µM of TJ191 (i.e. a high apoptosis-inducing dose) for two or nine days and thereafter TβRIII expression on the remaining live cells was analyzed. While there was no notable increase in TβRIII expression in PC3 cells, there was a significant increase in TβRIII expression in CEM cells after two and nine days of drug exposure (Figure 3C and 3D). Thus, TβRIII seems to be a marker for TJ191 sensitivity in T-cell leukemia/lymphoma cells, but not in cell lines derived from prostate cancer.

### TβRIII suppression does not correlate with sensitivity of conventional chemotherapeutics in malignant T cells

**Figure 4:**
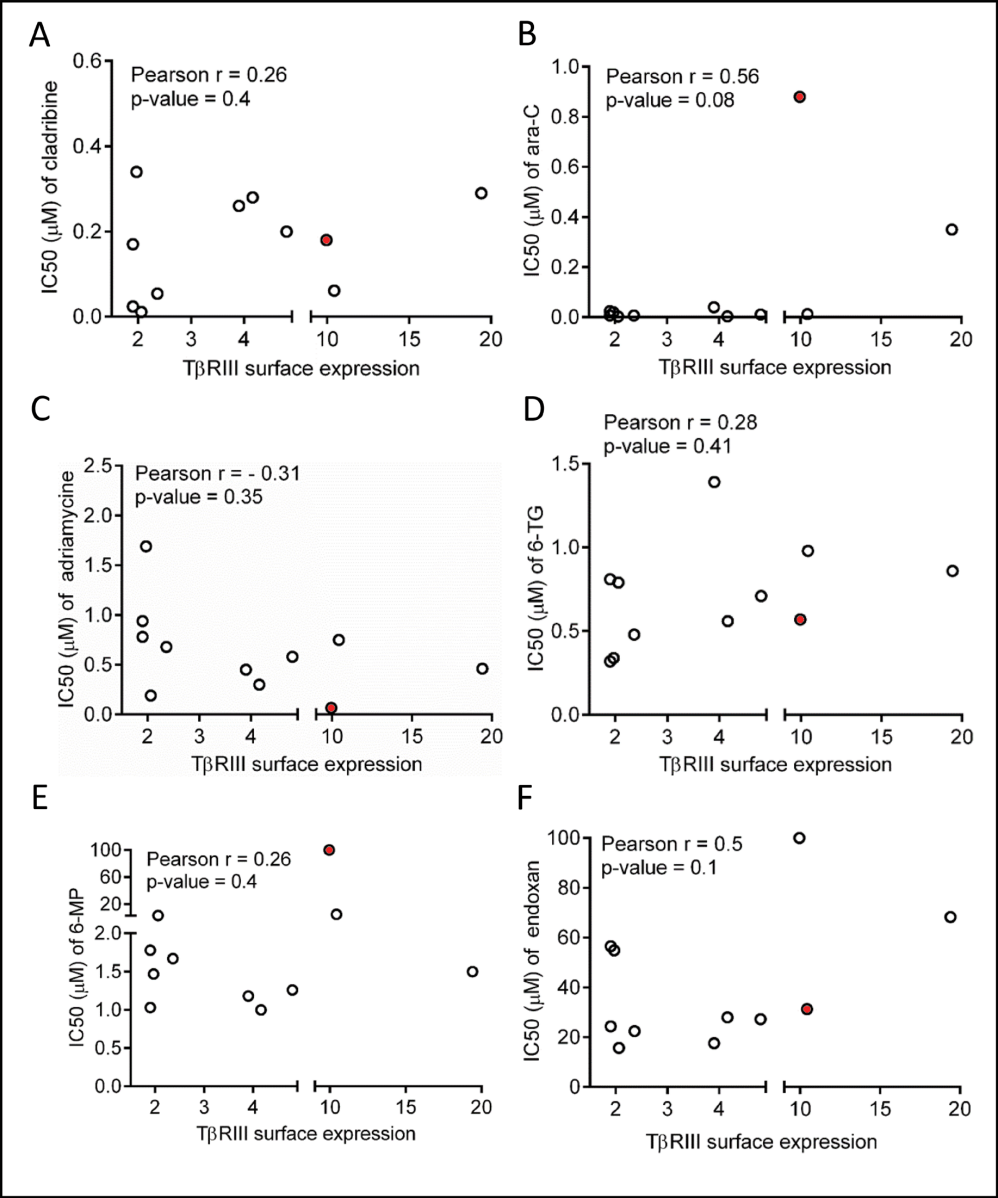
Lack of correlation between TβRIII expression and sensitivity of conventional chemotherapeutics in malignant T-cells (**A**–**F**) The IC_50_’s of cladribine, 6-mercaptopurine (6-MP), 1-β-D-arabinofuranosyl cytosine (ara-C), 6-thioguanine (6-TG), adriamycine and endoxan were correlated with surface TβRIII expression in the same panel of T-cell leukemia/lymphoma cell lines and PBMCs described previously in Figure 2E and 2F. There was no significant correlation between the IC_50_’s of all tested drugs and surface TβRIII expression levels. Correlations were detected using the Pearson product-moment correlation coefficient. Data shown were collected from three independent experiments. PBMC are indicated in red.

To confirm the specificity of the inverse correlation between TJ191 antiproliferative activity and TβRIII expression in T-cell leukemia/lymphoma cell lines, we examined whether the prognostic significance of TβRIII also exists for conventional chemotherapeutics that are currently used in treatment schedules of leukemia and lymphoma using the same panel of T-cell leukemia/lymphoma cell lines and multiple PBMC samples. Therefore, the IC_50_ values of cladribine, 6-MP, ara-C, 6-TG, adriamycine and endoxan were determined in selected cell lines and PBMCs and correlated with the TβRIII surface expression level. We found that the TβRIII surface expression level was not correlated with the IC_50_ of any of these chemotherapeutic agents (Figure 4). In contrast to TJ191, PBMCs were sensitive to most of the tested drugs, including cladribine (IC_50_ = 0.18 ± 0.09 µM), ara-C (IC_50_ = 0.88 ± 0.6 µM), 6-TG (IC_50_ = 0.57 ± 0.27 µM) and adriamycine (IC_50_ = 0.079 ± 0.062 µM). These results underscore the specificity of the inverse association between TβRIII expression and TJ191 sensitivity in T-cell leukemia/lymphoma cells. In addition, these findings indicate that the mechanism of action of TJ191 is not similar to any of the established anti-leukemia/lymphoma drugs.

### CRISPR/Cas9-mediated knock-out of TβRIII sensitizes malignant T-cells to TJ191-mediated cytostatic effects

**Figure 5:**
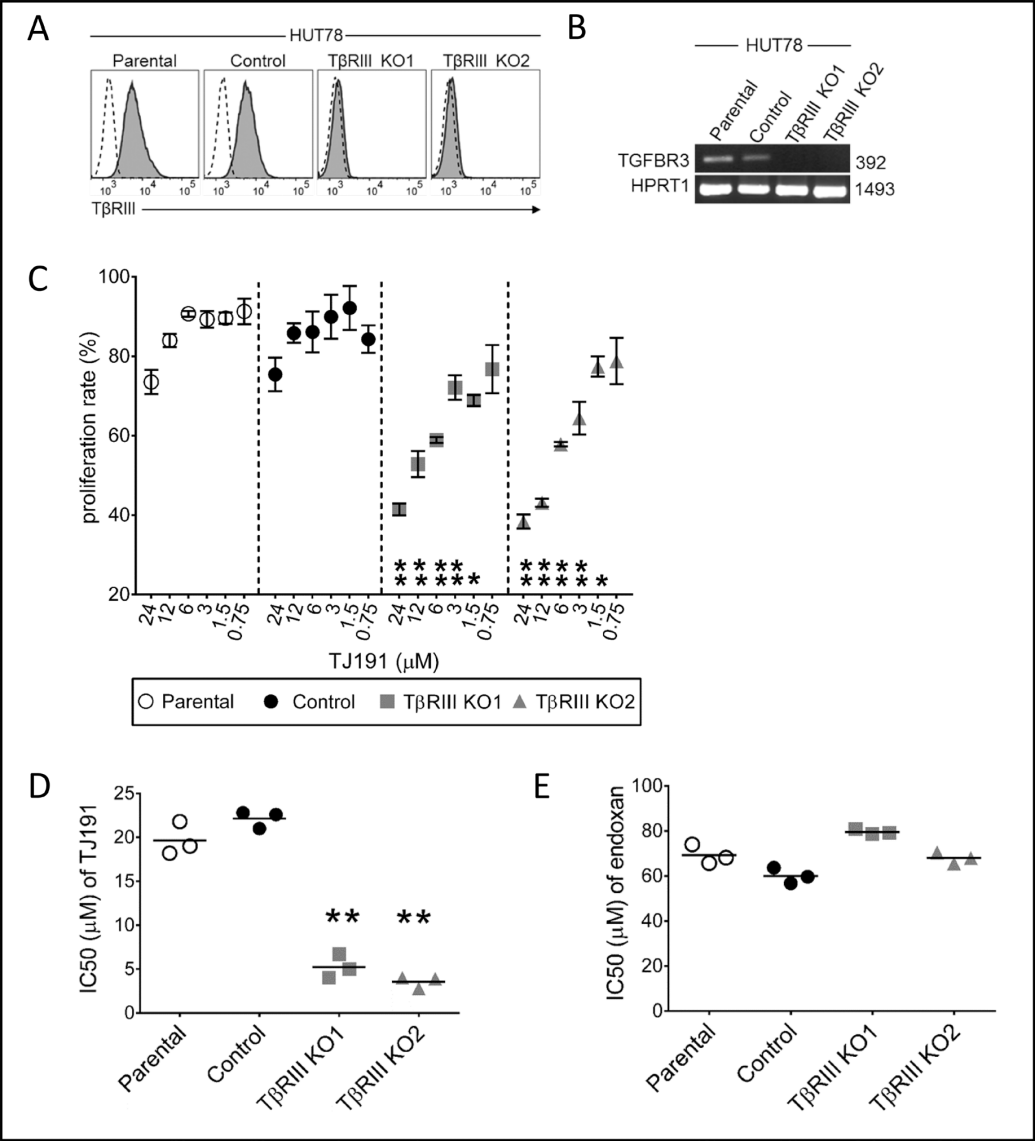
TβRIII knock-out by CRISPR/Cas9 approach enhances the susceptibility of T-cell lymphoma cells insensitive to TJ191 (**A**) Flow cytometry analysis of surface TβRIII expression in either parental, CRISPR/Cas9 scrambled monoclonal control or TβRIII knock-out from two different monoclonal (KO1 and KO2) insensitive cell lines (IC_50_ = 17 ± 10 µM). Dashed black lines in unfilled profile represent Ig isotype control staining and grey filled profiles indicate TβRIII staining. (**B**) Genomic DNA of parental, control, or TβRIII knock-out (KO1 and KO2) HUT-78 cells was isolated and TGFBR3 and HPRT1 were amplified by PCR. 31 cycles of PCR were performed and equal aliquots of each sample were loaded on a 1% agarose gel. (**C**) Dose-dependent effects of TJ191 against HUT-78 and its derivative cell lines. 1 × 10^4^ HUT-78 parental, CRISPR/Cas9 scrambled control, or TβRIII KO cells were treated with the specified concentration of TJ191. After three days, cells were counted and the rate of proliferation was measured. Symbols in each group show the absolute proliferation rate, calculated by subtracting the counted cells from the start cell number relative to untreated cells, at the indicated concentrations of TJ191. Data are the mean ± S.E.M of triplicates and are representative of three independent experiments. The asterisks indicate statistical significant difference ^*^p < 0.05 or ^**^p < 0.005, as determined by ANOVA at concentrations of 24, 12, 6, 3, and 1.5 µM obtained by comparing TβRIII KO1 or TβRIII KO2 with CRISPR/Cas9 scrambled control. (**D** and **E**) IC_50_ of D) TJ191 and (E) Endoxan in HUT-78 and its derivative cell lines was determined as indicated above. Data are representative of two independent experiments, each performed in triplicate. The line inside each group represents the mean of triplicate. The asterisks indicate statistical significant differences (^**^p < 0.005) as determined by ANOVA.

To determine whether TβRIII acts only as a predictive marker for TJ191 sensitivity of the cells or whether it is also involved in regulating the cytostatic activity of TJ191, we applied CRISPR/Cas9 technology to knock-out (KO) TβRIII in human TJ191-resistant HUT-78 cells (IC_50_ = 17 ± 10 µM). To evaluate the specificity of the CRISPR/Cas9 system to suppress TβRIII, we used the control scrambled CRISPR/Cas9 plasmid. Monoclonal cells were generated in both scrambled CRISPR/Cas9 control and TβRIII KO cells. Sequencing of *TGFBR3* genomic DNA, and flow cytometric analysis of TβRIII surface expression level confirmed that TβRIII is specifically knocked-out in KO1 and KO2 HUT-78 cells, but not in scrambled control or parental cells (Figure 5A and 5B). Consequently, parental and genetically manipulated HUT-78 cells were treated with TJ191 at different concentrations and cellular proliferation was assessed. The proliferation rate was significantly reduced in drug-exposed TβRIII KO1 and KO2 cells as compared to parental or scrambled control cells (Figure 5C). The IC_50_ values were indeed reduced at least four- to five-fold in TβRIII KO1 and KO2 cells compared with parental or scrambled control cells (Figure 5D). To investigate whether TβRIII KO also alters T-cell leukemia/lymphoma sensitivity to other chemotherapeutic drugs, HUT-78 wild-type and KO cells were treated with endoxan, which showed resistance among the tested drugs (Figure 4). In contrast to TJ191, no significant difference in endoxan sensitivity was observed between parental, scrambled control and TβRIII KO1 and KO2 cells (Figure 5E). Collectively, these data indicate that TβRIII plays a fundamental role in predicting and possibly regulating the selective cytostatic activity of TJ191 in T-cell leukemia/lymphoma cells.

## DISCUSSION

Carcinogenesis is a complex multistep process during which cells accumulate (epi)genetic alterations that eventually yield a malignant state. In the past decades, there has been a tremendous increase in our knowledge of the molecular mechanisms that trigger and fuel tumor growth and invasion. Cancer cells are capable of inducing multiple cellular processes, by activating various signaling cascades. Accordingly, new agents have been developed that block pathways, which are specifically activated in cancer cells. However, highly specific drugs that target a specific pro-tumor factor are not widely applicable. Moreover, tumors may escape this therapy by switching to other tumor-promoting pathways. Therefore, most chemotherapy regimens still include non-specific cytotoxic drugs, which cause systemic toxicity and often lead to tumor recurrence and treatment failure [2, 4]. Thus, a new generation of more specific cytotoxic agents is urgently needed.

We recently reported the synthesis and anti-proliferative activity of TJ191, a novel selective anti-cancer compound that targets various tumor cell types without affecting normal fibroblasts or immune cells [18]. Data collected from over 70 cancer cell lines (i.e. the NCI60 human tumor cell line panel and our in-house tumor cell lines) revealed that about 30% of all tumor cell lines are sensitive to the antiproliferative activity of nanomolar concentrations of TJ191 and that T-cell leukemia/lymphomas present the highest antiproliferative response rate to TJ191 among the tested cancer types. Therefore, we now focused our analysis on T-cell leukemia/lymphoma and found that TJ191 not only elicits cytostatic activity, but that it also induces apoptosis in sensitive human T-leukemia CEM cells in a time- and dose-dependent fashion.

Moreover, the antiproliferative dose-response produced by TJ191 in 60 cell lines of the NCI60 screen showed a tumor cell-selectivity pattern that was not found for any of the standard prototype compounds included in the NCI60 database [19]. This indicates that the response pattern of tumor cells to TJ191 is unique and that it may have a novel molecular mechanism of action. To explore the mechanism responsible for the cytostatic/cytotoxic activity of TJ191 in T-cell leukemia, we first selected resistant variants of the drug-sensitive CEM cells (CEM-R) in order to reduce genomic differences between naturally sensitive and insensitive cancer cell lines. Using RNA-seq to compare the gene expression profile of CEM and CEM-R cells, we found that TβRIII was upregulated in CEM-R cells. Validation of these findings in other T-cell leukemia/lymphoma cells and PBMCs revealed that TβRIII expression indeed inversely correlates with TJ191 sensitivity in T-cell leukemia/lymphoma cell lines. No such correlation was found with traditional chemotherapeutic drugs that are currently used clinically for the treatment of leukemia/lymphoma, such as cladribine, ara-C, adriamycine, 6-TG, 6-MP and endoxan. This inverse correlation was also not observed in a different cancer type, such as prostate cancer. We thus propose that TβRIII may serve as a useful biomarker to identify T-cell leukemia/lymphomas that are sensitive to TJ191.

Although TβRIII has been shown to play an essential role in tumor development, its role in T-cell malignancies is largely unknown. To assess the effects of complete loss of TβRIII on the antiproliferative activity of TJ191 in malignant T cells, we applied CRISPR/Cas9 technology to naturally drug-insensitive TβRIII-expressing HUT-78 cells. TβRIII knock-out promoted sensitization of the cells to TJ191, suggesting that TβRIII affects the antiproliferative effects of TJ191.

Although these data are novel and interesting, several questions remain to be answered. First, whereas parental HUT-78 cells (IC_50_ = 17 ± 10 µM) are 130-fold less sensitive to the antiproliferative activity of TJ191 than CEM cells (IC_50_ = 0.13 ± 0.02 µM), knocking out TβRIII expression in HUT-78 only partially increased the sensitivity of the resulting cell line. This indicates that the effect of TβRIII on TJ191 sensitivity is more complex than anticipated based on the correlation data (Figure 3). Alternatively, although both of T-cell origin, CEM and HUT-78 cells are largely different. CEM cells originate from a child with acute lymphoblastic leukemia (T-ALL), whereas HUT-78 cells are derived from a cutaneous T-lymphocyte in an adult male. Consequently, these cell lines are differentially regulated and modulation of one single receptor might have diverse outcomes depending on the cell line. Moreover, TβRIII is known to play various roles in cancer progression, and various factors, such as the cell context can determine its activity, which may even more complicate data interpretation.

Second, since TβRIII is mainly expressed on TJ191-resistant cells but not on -sensitive cells, this surface receptor is likely not the direct interacting molecular target of TJ191. Studies, including a positive selection screen using a CRISPR library, are ongoing to identify the molecular target of TJ191. Target identification would generate new insights in the antiproliferative/cytotoxic action of TJ191, and will also allow further optimization of its structure to ensure improved target inhibition and tumor suppression.

Third, and related to the previous point, a limitation to the use of TJ191 is its relative water insolubility, which precludes *in vivo* testing. To circumvent this problem, efforts are ongoing to develop prodrugs of TJ191 with improved solubility while retaining selectivity. In the past we successfully improved the solubility of antiviral and anticancer drugs by conjugation with L-Ser as in AVE-8062 or with an L-Lys-L-Pro dipeptide as in TUB091, which is released by the ubiquitous enzyme dipeptidyl peptidase IV (DPP-IV/CD26) in the *in vivo* setting [20–22].

Fourth, it is not clear so far whether the enhanced TβRIII expression upon exposure of CEM cells for 2 or 9 days to TJ191 (Figure 3C) results from selection of a pre-existing TJ191-resistant tumor cell fraction already present in the tumor cell culture or from de novo generation and selection of a mutant TJ191-resistant phenotype under the continuous pressure of TJ191. In any case, resistance development of TJ191-treated leukemia/lymphoma will most likely occur upon prolonged drug exposure, as it occurs also for other cancer drugs. Therefore, TJ191 should preferentially be combined with other established cancer drugs to afford an optimal anticancer activity.

Finally, elucidation of the molecular target would also permit the synthesis of totally different molecular structures, taking into account solubility as well as drug resistance issues.

Overall, our findings strongly indicate that TJ191 may be a valuable prototype compound in personalized cancer therapy and particularly for patients with T-cell leukemia and lymphoma that display low TβRIII expression levels.

## MATERIALS AND METHODS

### Drugs

Cladribine (kindly provided by the late Prof. Chris McGuigan, Cardiff University, UK), 6-mercaptopurine (6-MP, Sigma-Aldrich, Overijse, Belgium), 1-β-D-arabinofuranosyl cytosine (ara-C, Upjohn, Puurs, Belgium), 6-thioguanine (6-TG, Sigma-Aldrich), adriamycine (Sigma-Aldrich), and endoxan (cyclophosphamide, Janssen-Cilag, Beerse, Belgium) were used as reference compounds. TJ191 was synthesized as described [18].

### Primary cells and cell lines

PBMCs were isolated from Buffy coats obtained from healthy blood donors (after receiving informed consent) using lymphoprep according to the manufacturer’s instructions. PBMCs were then stimulated with interleukin-2 (2 ng/ml, R&D systems, Abingdon, UK) and phytohemagglutinin (2 µg/ml, Sigma-Aldrich) for three days and used in the experiments described below. Human T-cell leukemia/lymphoma cell lines CCRF-CEM (referred to as CEM), JURKAT, MOLT-3, MOLT-4, SUP-T1, MT-2, MT-4, C8166, HSB-2, and HUT-78 were obtained from ATCC (Middlesex, UK). Cells were cultured in RPMI-1640 medium supplemented with 10% fetal bovine serum (FBS, Gibco, Erembodegem, Belgium) and 2 mM L-glutamine (Gibco).

Human prostate cancer cell lines PC3, BPH-1, DU-145, PNT1A, 22RV1 and LNCAP were cultured as described in [16].

### Proliferation assays

Human T-cell leukemia/lymphoma and prostate cancer cell lines were seeded into 96-well plates at a density of 60 × 10^3^ and 20 × 10^3^ cells/well respectively, and treated with 5-fold dilutions of TJ191, cladribine, 6-MP, ara-C, 6-TG, adriamycine or endoxan. 200 × 10^3^ PBMCs/well were seeded and treated as indicated above. After three days, cells were counted using the coulter cell counter (Analis, Belgium). The IC_50_ value refers to the compound concentration that causes 50% inhibition of cell growth.

### Apoptosis assays

6 × 10^5^ CEM cells/well were seeded into 96-well plates and treated with TJ191 in the presence of 2 µM NucView 488 caspase substrate (Biotium, Hayward, CA). When cells undergo apoptosis, the caspase substrate is cleaved by activated caspase-3/7 to release the DNA dye NucView 488, which stains the nucleus with bright green fluorescence. Cells were fixed at indicated times in 2% paraformaldehyde for 10 min at room temperature and analyzed for NucView 488 staining using a Carl Zeiss Axiovert 200 M inverted microscope (Zeiss, Göttingen, Germany).

### qRT-PCR, PCR, and genomic DNA sequencing

To analyze relative expression of TβRIII in human T-cell leukemia/lymphoma and prostate cancer cell lines, TβRIII and HPRT1 (used for normalization) sequences were derived from RT-PCR amplicons of mRNA of selected cancer cell lines. qRT-PCR was performed in triplicate using GoTaq Sybr Green Master Mix (Promega, Leiden, The Netherlands) and specific primers in 96-well plates in a 7500 Fast Real-time PCR system (Applied Biosystems, Erembodegem, Belgium). Primers (forward and reverse primer sequences are listed 5′–3′) for *TGFBR3* are CCCAGTTCTTGTTCAGCCTTA, ATCACCTTCAACATGGAGCTAT and for HPRT1 are GCGATGTCAATAGGACTCCAG, TTGTTGTAGGATATGCCCTTGA. Relative expression of TβRIII was calculated from the threshold cycles (Ct) obtained with the 7500 Software Download v. 2.0.6 Software (Applied Biosystems).

For sequencing of *TGFBR3*, genomic DNA was isolated using QIAamp DNA mini kit (Qiagen, Antwerp, Belgium) according to the manufacturer’s instructions and sequenced at the KU Leuven genomic core facility. Primer sequences for exon 3 are (forward and reverse listed 5′-3′): TCTGAGTGGCTTGTGTTTGT, GAAATCCTAAAGTTGCCCAATCC and for exon 14: AGCTGTGTGCTGTCTGATAAA, GCAAGGGAGAGTGACCTTATG. PCR to analyze *TGFBR3* knock-out was performed using genomic DNA. Primers for exon 3 of *TGFBR3* are ATTGCTGTTTCTGAGTGGCT, CCTGCAGTGCGGAGATTC; and for HPRT1 are GCGATGTCAATAGGACTCCAG, TTGTTGTAGGATATGCCCTTGA. PCR conditions were 30 cycles at 94°C (denaturing, 15 seconds), 55°C (annealing, 30 seconds) and 72°C (extending, 1 min).

### Flow cytometry analysis

To analyze surface TβRIII expression on cancer cells and PBMCs, aliquots of 1 × 10^6^ cells were incubated with fixable viability stain 510 (FVS510, BD Biosciences, Erembodegem, Belgium) to discriminate viable from non-viable cells for 15 min, followed by centrifugation and two washes with PBS. Cells were then stained with PE anti-human TβRIII polyclonal antibody (LSBio, Huissen, The Netherlands). Flow cytometric analysis was performed on a BD FACSCanto II flow cytometer. Data were analyzed using the FlowJo software (Tree Star).

### Knock-out of TβRIII by CRISPR/Cas9

The TβRIII CRISPR/Cas9 KO-GFP plasmid system (Santa Cruz Biotechnology, Cambridge, UK) was used to knock-out TβRIII. For evaluating the specificity of the CRISPR/Cas9 system, we used the control scrambled CRISPR/Cas9-GFP plasmid (Santa Cruz), which encodes 20 nucleotides scramble guide RNA (gRNA) that does not target any gene in the human genome. TJ191-resistant HUT-78 cells were transfected by Neon transfection system (ThermoFisher, Erembodegem, Belgium) and transfectants were sorted for positive GFP expression using BD FACSAria and BD Diva software. GFP-positive cells were used to establish single cell clones of the selected cell line by limited dilution. The efficiency of CRISPR/Cas9 constructs in single cell clones was confirmed by sequencing of *TGFBR3* genomic DNA (isolated by using the Qiagen kit and sequenced at the KU Leuven genomic core facility) and measuring the surface TβRIII expression level using flow cytometry. Flow cytometry analysis was performed as described above using anti-TβRIII polyclonal antibody.

### Statistical analysis

To detect statistically significant differences between study groups and controls, two-sided student’s *t*-tests were applied. Where appropriate, a one-way Analysis of Variance (ANOVA) was used. To determine differences between individual variables, the Pearson product-moment correlation coefficient was used. All reported *p*-values were two-sided, *p*-values below 0.05 were considered as statistically significant and *p*-values below 0.005 were considered as highly significant. For the statistical evaluation GraphPad Prism6 was used.
